# Pooled Time Series Modeling Reveals Smoking Habit Memory Pattern

**DOI:** 10.3389/fpsyt.2020.00049

**Published:** 2020-02-19

**Authors:** Jesús F. Rosel, Marcel Elipe-Miravet, Eduardo Elósegui, Patricia Flor-Arasil, Francisco H. Machancoses, Jacinto Pallarés, Sara Puchol, Juan J. Canales

**Affiliations:** ^1^Department of Evolutionary Psychology, Educative, Social Studies and Methodology, Universitat Jaume I, Castellón, Spain; ^2^Department of Basic and Clinical Psychology and Psychobiology, Universitat Jaume I, Castellón, Spain; ^3^Department of Theory and History of Education and Methods of Research and Diagnosis in Education, Universidad de Málaga, Málaga, Spain; ^4^Department of Health Science, Valencian International University, Castelló de la Plana, Spain; ^5^Predepartmental Unit of Medicine, Universitat Jaume I, Castellón, Spain; ^6^IATS, Consejo Superior de Investigaciones Científicas, Castellón, Spain; ^7^School of Psychological Sciences, University of Tasmania, Hobart, TAS, Australia

**Keywords:** tobacco, pooled time series, panel time series, intensive data analysis, memory, multilevel regression

## Abstract

Smoking is a habit that is hard to break because nicotine is highly addictive and smoking behavior is strongly linked to multiple daily activities and routines. Here, we explored the effect of gender, age, day of the week, and previous smoking on the number of cigarettes smoked on any given day. Data consisted of daily records of the number of cigarettes participants smoked over an average period of 84 days. The sample included smokers (36 men and 26 women), aged between 18 and 26 years, who smoked at least five cigarettes a day and had smoked for at least 2 years. A panel data analysis was performed by way of multilevel pooled time series modeling. Smoking on any given day was a function of the number of cigarettes smoked on the previous day, and 2, 7, 14, 21, 28, 35, 42, 49, and 56 days previously, and the day of the week. Neither gender nor age influenced this pattern, with no multilevel effects being detected, thus the behavior of all participants fitted the same smoking model. These novel findings show empirically that smoking behavior is governed by firmly established temporal dependence patterns and inform temporal parameters for the rational design of smoking cessation programs.

## Introduction

Patterned behavior can be predicted on the basis of the behavior displayed in the immediate, or even remote, past. However, very few longitudinal studies in psychology and health sciences have used this approach to characterize specific behaviors, problematic or otherwise, although several authors have highlighted the usefulness of this kind of methodology (1–4).

We propose here that habitual smoking builds a temporally distinct smoking “memory”, defined, from a point of view of statistical time series, as the maximal distance (lags) from which the values of the series can be confidently predicted. For example, if a temporary process was measured in days, and the maximum delay to accurately forecast the series was five lags, it would follow that the memory of the series, this is, the autoregressive process (*AR*), is 5 days (*AR(5)*). This statistical concept is increasingly used in time series as applied in physical, social, engineering, and statistical sciences (5–8). Indeed, the designers of time series analysis have used this notion to define short and long memory processes (9). Our aim here is to temporally define the smoking habit memory pattern, understood as a statistical modeling of habitual smoking, and determine the maximum delay (behavioral memory) of the model.

Although the number of longitudinal studies has risen in the last 15 years (2, 4), very little research involving pooled time series (i.e. panel analysis, or different subjects measured several times over time) provides evidence that nicotine dependence is a function of smokers' *AR* memory. Three models have been proposed to explain the possible relationships between nicotine and the smoking habit (10). Velicer et al. (11) later reviewed the three models and assigned a time series model to each of them (11). The first model was the “nicotine fixed-effect” model, which has positive *AR* coefficients. A second model was the “nicotine regulation model,” which assumes a “zero” autocorrelation. The third model was the “multiple regulation model,” where the levels of smoking would fluctuate so that a reduction in smoking would give rise to a greater desire to smoke, which has negative *AR* coefficients.

The study by Velicer et al. (11) is one of only two studies in the literature that are similar to the present research. Velicer et al. (11) worked with a sample of ten smokers who recorded data twice a day for 62 days. In the other study (12), a sample of 29 participants was used, who recorded the number of cigarettes smoked over a period of 84 days. These previous studies showed that the memory for smoking spans 1 or 2 days, in one case, and 7 and 14 days, in the other. It is worth noting, however, that Velicer et al. (11) did not analyze the data in search for weekly seasonality, unlike Rosel and Elósigui (12), which can impact on the number of significant lags that are found.

The analyses performed to date on smoking habits have been mostly of a univariate nature, this is, they include only one person per analysis and, as established by Makridakis (13) and Box and Jenkins (14), have little statistical (9). The statistical power of a test is the probability that the test rejects the null hypothesis when the specific alternative hypothesis is true (15), so if a statistical test has low power (as in univariate times series estimation of parameters) it is more difficult to obtain significant results. The statistical power increases, however, with the use of pooled time series analysis (16–24).

From a cognitive approach to the study of addiction habits, there are two models of behavior subject to regularities. The first model includes components that allow it to be considered an incremental, persistent habit, or a “model-free system,” that is largely insensitive to outcome devaluation and, in this case, the *AR* coefficients will all be positive. However, if the behavior also involves evaluation and decision making directed toward achieving certain objectives, such as trying to reduce the number of cigarettes smoked per day, or quitting the habit, then the behavior would be an example of a “model-based system” (25–29). In time series this implies that they will present one or several negative *AR* coefficients.

### Hypotheses

Several hypotheses were postulated for this study. The main hypothesis was that the smoking habit is expected to have an *AR* component with respect to previous days (9, 30). In other words, the number of cigarettes smoked on any given day will be a function of the number of cigarettes smoked on previous days, but could also vary according to weekly memory patterns^1^. Please note we have removed the equations from the main text and included them as footnotes to facilitate readability for readership not specialized in time series.

Similarly, it is assumed that the values of the coefficients will be positive in accordance with the nicotine fixed-effect model (11) or with the model-free system (25, 28, 29).

The data collection model had two levels. The first refers to the number of cigarettes smoked daily, while the second level is the person, and thus each daily item of data belongs to a particular smoker (31–38). Hence, the hypothesis here is that the coefficients *b*_0_, *b*_1_ of the lag *Y*_t−1,j_ and *b*_7_ of the lag *Y*_t−7,j_ differ from one participant to another^2^.

It is also hypothesized that the number of cigarettes smoked is a function of the day of the week, for which six dummy variables were created, taking Sunday as the reference^3^.

We tested whether the variables gender and age influenced the number of cigarettes smoked each. Both are level 2 variables because they have a fixed value for each person, this is, these values do not change for each of the participants (*γ*_0_), but there is a random component for each individual (*u_Gj_*)^4^.

**Figure 1:**
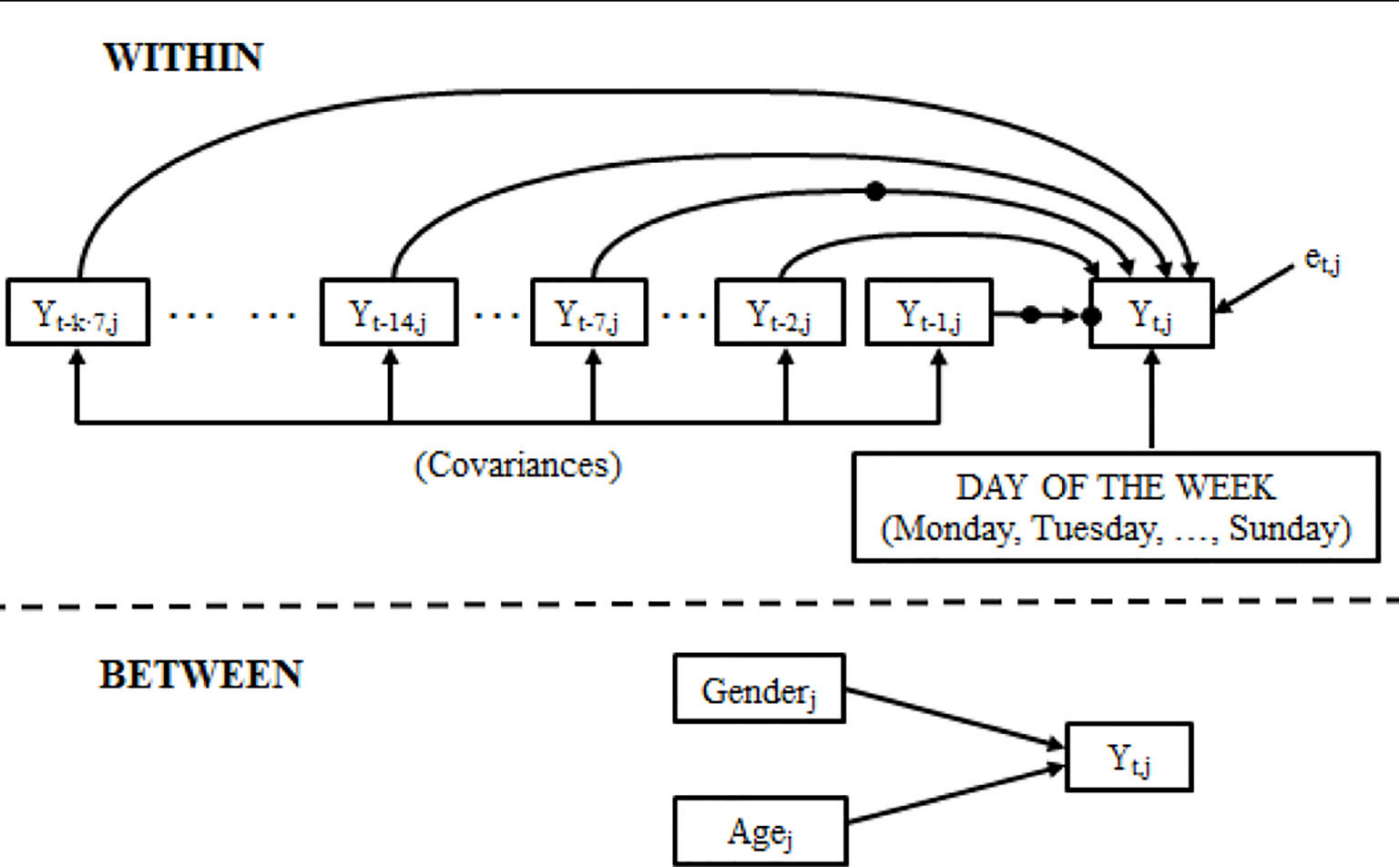
Representation of the hypothesized model. The highlighted points indicate random coefficients.

The hypotheses with the final model can be expressed with the following equation^5^ and are shown in a simplified manner in **Figure 1** (39).

We have included hypothetical *constraints* for the data analysis, which were laid out in order to simplify the model in coherence with it being a time series. The constraints were:

- the greater the elapsed time, the smaller the non-seasonal *time effects* are for the days immediately prior to the measurement of Y_t_ (Y_t−1_, Y_t−2_, …); this is, the greater the time interval between the moment of the lagged measurement (Y_t-j_) and the moment of the forecast (Y_t_), the smaller the temporal memory of the system will be. Likewise, the greater the amount of time elapsed, the less effect of the weekly seasonality (Y_t−7_, Y_t−14_, Y_t−14_, …, Y_t−7·k_) will have on Y_t_ (9, 40)^6^.
- equality of the *variances* of the temporal independent variables, since the same lagged variable is used^7^.
- the *covariances* between the pairwise temporal independent variables are assumed to be equal, based on the distance (in days) between the lags^8^.

## Method

### Participants

Smokers were first- or second-year students of the Psychology and Child Education degrees of the University of Málaga (Spain). Participation in the study was voluntary. The research was carried out with the approval of the University of Málaga Human Ethics Committee, and each participant signed an informed consent document prior to taking part in the study. In order to be included in the sample participants had to meet three requirements: (i) they must have been smoking for more than two consecutive years prior to data collection, (ii) they had to smoke more than five cigarettes a day, and (iii) they had to fill in a smoking record sheet for at least 63 consecutive days. Participants were not screened for health conditions, including psychological or neurological disorders. Of the 310 students that expressed an interest in the study, 99 (31.9%) stated that they were habitual smokers.

Finally, 80 students took part in the study. However, 13 of them did not submit fully completed records, and 5 participants were excluded because they smoked an average of fewer than five cigarettes per day during the study. The final study sample therefore consisted of 62 participants, of whom 36 were male and 26 were female (57.1% and 42.9% of the final sample, respectively). The participants were between 18 and 26 years of age, the mean being 20.44 years for the male, 20.37 years for the female and 20.41 years for the sample as a whole. A sample of 62 participants is considerable, generating a total sample with a chain of 5159 longitudinal data.

### Procedure

Each participant recorded the number of cigarettes smoked by means of a daily self-report chart that was filled in each night. Recordings took place over a period that ranged between 69 to 91 days, the mode being 84 days, as 46 participants submitted a record of 84 days (74.2%).

A total of 5159 daily smoking data items were submitted but some items, 1.08% of the total, were missing. We did not estimate missing values because statistical systems for estimating missing values are designed for cross-sectional data, not longitudinal data.

### Data Analysis

The data for the analysis in this research were transcribed and entered in a statistics program (41). Descriptive analyses were also performed in the same program. Once the data had been introduced with a “person-level data set” system, they were transferred to a “longitudinal time record” system (42), each record corresponding to a day of smoking, and were organized for analysis with a pooled time series system (17, 43, 44). After organizing the data, they were analyzed using the statistics software package Mplus (39). The value α = .05 was taken as the level of significance. Data, input, and output files in Mplus can be obtained from this website: http://repositori.uji.es/xmlui/handle/10234/180682.

## Results

**Table 1** shows the mean and the SD of the number of cigarettes smoked by the participants every day of the week. The day on which the participants in the sample smoked the least was Monday, the figure rising slowly day by day until Saturday, which was the day they smoked the most. The mean of the number of cigarettes smoked per day by the participants was 15.664 (*SD* = 8.257). Levene's test was performed (*F* (1, 46) = 1.187, *p* = .310) on the number of cigarettes smoked daily as a function of the day of the week. The assumption of homogeneity of the variances was met.

**Table 1A:** Means and SD of the number of cigarettes smoked by the sample.

Measure	Day of the week						
	Monday	Tuesday	Wednesday	Thursday	Friday	Saturday	Sunday
Mean	14.38	15.03	15.11	15.03	16.99	17.62	15.46
SD	8.35	8.06	7.88	8.07	8.29	8.42	8.25

**Table 1B:** Participants ordered according to their direct and homoscedastic mean and SD, from lowest to highest.

Participant	Raw data	Homoscedastic data
	Y_t,j_	${\text{Y}}_{\text{t,j}}^{\text{H}}$
	M (SD)	M (SD)
18	5.56 (2.99)	1.86 (1.00)
35	5.79 (2.29)	2.53 (1.00)
19	6.21 (1.73)	3.59 (1.00)
1	6.27 (1.88)	3.34 (1.00)
–	–	–
45	26.11 (5.45)	4.72 (1.00)
51	30.57 (5.77)	5.29 (1.00)
71	35.13 (5.28)	6.656 (1.00)
24	42.11 (7.56)	5.567 (1.00)

The intraclass correlation (ICC) was .802. The consistency between the measures was above 75% and therefore it represented excellent intraclass reliability (45).

**Table 1** also shows the means and the SD of the four participants with the lowest mean and the four with the highest mean number of cigarettes smoked per day. Due to the large difference in the SD of the participants, we tested the homoscedasticity of the dependent variable. The result showed that the data were heterogeneous (*F*(61, 5097) = 291.04, *p* < .001), and therefore the variance of the series as a function of the subjects was not constant.

The systems for estimating parameters based on the normality of the residuals are not very efficient when there is no homogeneity of variances, as they can lead to type II errors, or failure to reject a false null hypothesis (46, 47). Additionally, because the subjects have different variances, the precision of the forecasts will be different depending on the individuals, thereby giving rise to variances of different magnitudes in the residuals. The decision was therefore made to transform each value of *Y*_t,j_ by dividing it by the corresponding standard deviation of each participant (*s*_j_).

(9) $$\frac{{\text{Y}}_{\text{t},\text{j}}}{{\text{s}}_{\text{j}}}={\text{Y}}_{\text{t},\text{j}}^{\text{H}}$$

By so doing, each raw value is converted into a new one, but now the variances of ${\text{Y}}_{\text{t,j}}^{\text{H}}$ are homogeneous and equal to unity: Var(Yh_t,j_|j) = Constant = 1. We will call the new transformed variable “homoscedastic number of cigarettes per day” (${\text{Y}}_{\text{t},\text{j}}^{\text{H}}$).

Note in the column ${\text{Y}}_{\text{t,j}}^{\text{H}}$ in **Table 1** that each participant now has a variance equal to one, the new homoscedastic mean is 4.546, the minimum homoscedastic mean is 1.860 and the maximum is 6.657, while the total variance of ${\text{Y}}_{\text{t},\text{j}}^{\text{H}}$ is 2.253, which is not equal to one because not all the subjects have the same mean.

In order to determine the maximum number of lags to be introduced into the model, the simple autocorrelation function and the partial autocorrelation function of the series of ${\text{Y}}_{\text{t},\text{j}}^{\text{H}}$ were calculated. It was found that the lags are significant up to 56 days, with a seasonal pattern being repeated every 7 days.

With regards to the estimation of the hypothesized model, Equation 5, which we will call Model 1 (M1), was tested (**Table 2**). Data showed that this model did not converge. Due to this, each of the level 2 independent variables were removed one by one: gender and age (since the estimation did not converge), and each of the random coefficients were also removed separately from the intercept, from lag 1 and from lag 7. Even so, the estimation model did not converge or was not significant. The fixed part of the model was left, and each of the level 2 variables (gender and age) and each of the random coefficients from Equations 2 and 4 were introduced separately, although it still failed to converge. The overall fit indicators of each estimated model are shown in **Table 2**.

**Table 2A:** Summary of the models.

Models	*df*	*X*^2^	*p*	RMSEA (90% CI^a^)	CFI	TLI	SRMR	AIC
M1	Does not converge							
M2	89	554.797	<.001	.056 (.051–.060)	.848	.973	.036	56002.439
M3	29	43.403	<.042	.017 (.003–.027)	.995	.998	.014	56016.744
M0^b^	136	51403.800	<.001	.355 (.353–.358)	.000	.000	.471	160384.664

^*a*^CI, Confidence Interval. ^b^M0, Null model with respect to M2.

**Table 2B:** Coefficients of the regression model, homoscedastic scores.

Variable	Estimate	*SE*	*t*	β(standardized)	*p*-value
Intercept	.2618	.078	3.348	.1164	.001^a^
${\text{Y}}_{\text{t}-1,\text{j}}^{\text{H}}$	.2468	.023	10.624	.2485	<.001^b^
${\text{Y}}_{\text{t}-2,\text{j}}^{\text{H}}$	.0983	.022	4.501	.0990	<.001^b^
${\text{Y}}_{\text{t}-7,\text{j}}^{\text{H}}$	.1934	.023	8.494	.1947	<.001^b^
${\text{Y}}_{\text{t}-14,\text{j}}^{\text{H}}$	.0867	.024	3.662	.0873	<.001^b^
${\text{Y}}_{\text{t}-21,\text{j}}^{\text{H}}$	.0716	.023	3.065	.0721	.001^b^
${\text{Y}}_{\text{t}-28,\text{j}}^{\text{H}}$	.0653	.016	4.179	.0657	<.001^b^
${\text{Y}}_{\text{t}-35,\text{j}}^{\text{H}}$	.0652	.016	4.173	.0656	<.001^b^
${\text{Y}}_{\text{t}-42,\text{j}}^{\text{H}}$	.0523	.011	4.792	.0526	<.001^b^
${\text{Y}}_{\text{t}-49,\text{j}}^{\text{H}}$	.0522	.011	4.783	.0525	<.001^b^
${\text{Y}}_{\text{t}-56,\text{j}}^{\text{H}}$	.0521	.011	4.778	.0524	<.001^b^
Day of the week (Δ(χ^2^) = 511.394, Δ(*df*) = 60)					<.001^a^ ^c^
D_Mon_	−.3845	.084	−4.563	−.0590	<.001^a^
D_Tue_	−.2052	.085	−2.403	−.0316	.016^a^
D_Wed_	−.1910	.084	−2.264	−.0299	.024^a^
D_Thu_	−.1479	.084	−1.765	−.0231	.078^a^
D_Fri_	−.0449	.087	−.519	−.0070	.604^a^
D_Sat_	−.0732	.086	−.847	−.0114	.397^a^

a Two-tailed probability.

b One-tailed probability, positive value.

c Calculated by means of the difference between M2 and M3.

Finally, only the level 1 variables (intercept, days of the week dummy and lags) were left, and this model was called model 2 (M2). **Table 2** shows the results of M2, where it can be observed that all the variables are significant at α = .05.

Additionally, in order to determine the exact probability of the overall variable day of the week, model 3 (M3) was estimated, which was made up of only the intercept and the *AR* values.

Thus, as M3 is nested in M2 (48, 49), the difference in χ^2^ is 511,394 (Δχ^2^ (M2,M3)), with a difference in the number of degrees of freedom (Δ*df*) of 60, which yields a *p* < .001. Thus, the variable day of the week is significant (p < .001). Likewise, in order to test the exact significance of the overall fit of M2, the null model (M0) of M2 was used as the reference model, this is, with all the M2 variables, but indicating that the covariances of all the variables are equal to zero. It was found that Δχ^2^ (M0,M2) = 50849.003, and that Δ*df* (M0,M2) = 47, which means that the probability of these differences is *p* < .001, indicating that the overall fit of M2 is significant.

Residuals (*e*_t,j_ values) must be normal, and they should not be autocorrelated. The normality of the residuals was tested by applying the Kolmogorov–Smirnov test (*p* = .064), and it can therefore be concluded that the residuals of M2 follow a normal distribution. To check that the residuals are “white noise” we applied the Ljung–Box test (50), the result of the χ^2^ for three seasons, or 21 lags, being 27.055 (*df* = 21, *p* = .169), which indicates that the residuals are indeed “white noise.”

**Figure 2:**
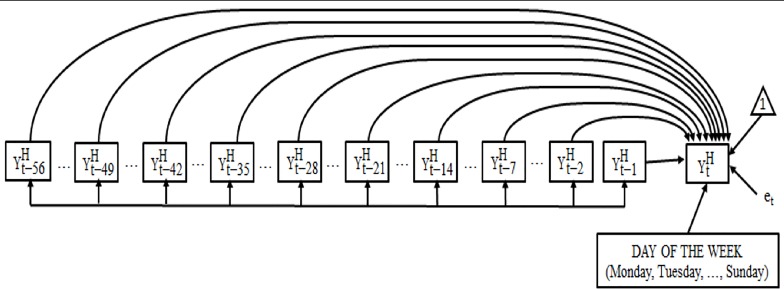
Representation of the final model obtained, in homoscedastic values.

Of all the models analyzed, we accept M2 as the model that best fits the data and our hypotheses because: (i) the overall indicators are good, except CFI, which is still acceptable, and, moreover, the test of the exact probability of the overall fit and the *R^2^* of the dependent variable are both significant (*R^2^* = .837, p < .001); (ii) the independent variables are significant; (iii) the AIC of M2 is lower than that of M3 (**Table 2**); and (iv) the residuals are normal and “white noise.” **Figure 2** show the results of M2.

## Discussion

The results obtained in this research confirm the main hypotheses of the proposed model, this is, that the smoking habit has an *AR* component, specifically of 56 days, and that the day of the week leads to a change in the number of cigarettes smoked. As stated in the introduction, previous studies found that participants followed *AR* models with a maximum of 14 days. In this study, however, we have found that participants follow a simple *AR* model of 2 days, to which we must add the seasonal pattern of 7 days with eight seasons, this is, a total of 56 days. This finding suggests that the number of cigarettes a person smokes on a given day is influenced by the day of the week and by the number of cigarettes smoked 1, 2, 7, 14, 21, and so forth up to 56, days before. This implies that the implicit memory for smoking spans 56 days. Furthermore, all the *AR* coefficients were positive, confirming the “nicotine fixed-effect model” (11), also referred to as the model-free smoking habit (27).

The fact that people smoke with a regular weekly pattern indicates the extent to which their habits are conditioned and subject to unwritten behavioral norms and other norms that are driven by social demands with respect to their routines, e.g. meeting up with friends to go out at the weekend and smoke. Likewise, when a person smokes, he or she incorporates different aspects of his or her behavior into daily life: physical behavior, behavior aimed at apparently reducing stress through smoking tobacco, and habits associated with tobacco (hours of smoking; other simultaneous, previous or subsequent consumptions, such as alcohol or coffee; smoking after certain meals; etc.).

In the present study all subjects presented the same statistical model of smoking, with daily and weekly components, suggesting that participants responded similarly to a constellation of personal (Pavlovian habits, abstinence syndrome, particular response to stress, etc.), contextual (break-time, drinking coffee or alcohol, after meals, etc.) and social (co-workers, friends who smoke, parties, holidays, etc.) stimuli (28, 29).

One of the possible reasons that may explain why the multilevel hypotheses of the model were not confirmed could be the actual memory of the data. Lagging the dependent variable significantly up to 56 days means that the data have a very high memory load, thereby reproducing the level of the y-intercept through the level of lags, and this would therefore mean that the model does not require a multilevel model for the y-intercept or for the coefficients of lags 1 and 7.

The findings of this research must be evaluated within the context of several methodological limitations. One of them is the generalizability or external validity, since the sample used consisted exclusively of university students, this is, mostly young people. Furthermore, the model obtained is the same for all the participants in our sample, regardless of the smoker's gender or age. Given that the sample age was between 18 and 26 years, it is possible that if we had taken a sample with more variability in age, this variable could have been significant. More broadly, the fact that the sample was composed of young adults (i.e. university students) limits the generalizability of the findings. As an additional drawback, it is difficult to carry out a direct comparison between our results and those of other studies because, first, few studies have been conducted on this topic and, second, different methods of data analysis were used.

If the memory for smoking is 56 days, we might wonder whether, from a biological point of view, plasticity changes occur in brain areas associated with habit formation, including the basal ganglia and the prefrontal cortex (27, 51) that sustain such mnemonic and behavioral processes. This warrants further investigation. The data presented here with regards to the timeframe that allows smoking behavior to be confidently predicted (i.e., 8 weeks), which underlies the presence of a temporally resilient memory of the smoking habit, combined with the elucidation of the underlying neurobiological processes involved, could potentially lead to the design of more effective smoking cessation interventions. Such interventions should take into consideration these temporal parameters. Indeed, previous studies have shown that for cessation of smoking to be more effective, patients must engage for at least eight weeks in some kind of smoking cessation program because otherwise they are more likely have a relapse in the 12 months following the start of the program (52, 53). The present findings would therefore be in agreement with the conclusions of these studies. Taken together, these findings suggest that healthcare professionals should plan therapies or treatments, with their associated follow-ups, to last no less than 8 weeks in order to achieve higher percentages of success in giving up the smoking habit and preventing relapses both during that time and in the following 12 months.

In summary, the present study shows a range of novel findings. First, we found significant results until lag 56, this is, more lags than in previous studies, indicating that a person's memory for smoking is longer-lasting than previously thought. Second, we observed that the day of the week also influences the smoking habit. Third, the multilevel hypotheses on the coefficients were not confirmed, and no differences in smoking habits were found according to gender or age, indicating that the behavior is common to all the individuals in the sample. Further research is needed to be able to generalize the results presented here and to apply them in future studies on prevention as well as treatment for smokers.

## Data Availability Statement

The datasets generated for this study can be found in the Repositori Universitat Jaume I at http://repositori.uji.es/xmlui/handle/10234/180682.

## Ethics Statement

The studies involving human participants were reviewed and approved by the University of Málaga Human Ethics Committee. The patients/participants provided their written informed consent to participate in this study.

## Author Contributions

EE collected the data. JR, ME-M, PF-A, FM, JP and SP analysed the data. JR, ME-M and JC wrote the manuscript.

## Conflict of Interest

The authors declare that the research was conducted in the absence of any commercial or financial relationships that could be construed as a potential conflict of interest.

## Footnotes

1 This could be expressed with the following equation:

(1) $$Y_{t,j}=f(Y_{t-1,j},Y_{t-2,j},...,Y_{t-7,j},Y_{t-14,j},...,Y_{t-7\cdot k,j}),$$

2 This could be expressed with the following set of equations:

$$b_{0j}=\gamma_{00}+u_{0j}$$

$$b_{1j}=\gamma_{10}+u_{1j}$$

(2) $$b_{7j}=\gamma_{70}+u_{7j}$$

where *γ_00_* would now be the fixed part of the intercept and is equivalent to the mean of the level 2 intercepts. The value *u_0j_* would be the random part of the second-level intercept of the participants in the sample, and there would be as many intercepts as there are participants. The same interpretation is assumed for *b_lj_* and *b_7j_*.

3 Being the codes:

(3) $$\text{Day of the week}=[D_{\text{Mon}},D_{\text{Tue}},D_{\text{Wed}},...,D_{\text{Sat}}]$$

4 Hence, if the coefficients for gender and age are *b_G,j_* and *b_Ed,j_*, their corresponding multilevel coefficients will be:

$$b_{G,j}=\gamma_{0G}+u_{G,j}$$

(4) $$b_{Ed,j}=\gamma_{0Ed}+u_{Ed,j}$$

the properties of the terms in Equation 4 being similar to those in Equation 2.

5 General equation with the hypotheses:

(5) $$Y_{t,j}=(\gamma_{00}+u_{0j})+(\gamma_{10}+u_{1j})\cdot Y_{t-1,j}+b_{2}\cdot Y_{t-2,j}+b_{3}\cdot Y_{t-3,j}+...+(\gamma_{70}+u_{7j})\cdot Y_{t-7,j}+b_{14}\cdot Y_{t-14,j}+...+b_{k\cdot 7}\cdot Y_{t-7\cdot k,j}+[b_{\mathrm{Mon}}\cdot D_{\text{Mon}}+b_{\text{Tue}}\cdot D_{\mathrm{Tue}}+...+b_{\mathrm{Sat}}\cdot D_{\mathrm{Sat}}]+(\gamma_{0G}+u_{G,j})\cdot \text{Gender}+(\gamma_{0Ed}+u_{Ed,j})\cdot \mathrm{Age}+e_{t,j},$$

6 Constraints on the coefficients

$$b_{1}>b_{2}>b_{3}>...(\text{for the simple AR components})$$

(6) $$b_{7}>b_{14}>...>b_{7\cdot k}(\text{for the }7-\text{day seasonal }\mathrm{AR}\text{ components})$$

7 Equations:

$$\mathrm{Var}(Y_{t-1})=\mathrm{Var}(Y_{t-2})=...=\mathrm{Var}(Y_{t-7})=$$

(7) $$\mathrm{Var}(Y_{t-14})=\mathrm{Var}(Y_{t-21})=...=\mathrm{Var}(Y_{t-7\cdot k})$$

8 Hence, for the covariances between successive days:

$$\mathrm{Cov}(Y_{t-1},Y_{t-2})=\mathrm{Cov}(Y_{t-2},Y_{t-3})=...=\mathrm{Cov}(Y_{t-h},Y_{t-h-1}).$$

For the covariances between each set of 7 days, 7·k being the last lag in the series:

$$\mathrm{Cov}(Y_{t-7},Y_{t-14})=\mathrm{Cov}(Y_{t-14},Y_{t-21})=\mathrm{Cov}(Y_{t-21},Y_{t-28})=...=\mathrm{Cov}(Y_{t-7\cdot k+7},Y_{t-7\cdot k}).$$

For the covariances between each set of 14 days:

$$\mathrm{Cov}(Y_{t-7},Y_{t-21})=\mathrm{Cov}(Y_{t-14},Y_{t-28})=...=\mathrm{Cov}(Y_{t-7\cdot k+14},Y_{t-7\cdot k}).$$

In the same way, the covariances with a distance of 21 days between each other have been equalised:

$$\mathrm{Cov}(Y_{t-7},Y_{t-28})=...=\mathrm{Cov}(Y_{t-7\cdot k+21},Y_{t-7\cdot k});$$

$$\begin{array}{ll} \text{between }28\text{ days}:\; & \text{Cov}({\text{Y}}_{\text{t}-7},{\text{Y}}_{\text{t}-35})=\ldots =\text{Cov}({\text{Y}}_{\text{t}-7\cdot \text{k}+28},{\text{Y}}_{\text{t}-7\cdot \text{k}}); \end{array}$$

(8) $$\begin{array}{ll} \text{and between }35\text{ days: } & \text{Cov}({\text{Y}}_{\text{t}-7},{\text{Y}}_{\text{t}-42})=\ldots =\text{Cov}({\text{Y}}_{\text{t}-7\cdot \text{k}+35},{\text{Y}}_{\text{t}-7\cdot \text{k}}). \end{array}$$
